# *In Vitro* Neurochemical Assessment of Methylphenidate and Its “Legal High” Analogs 3,4-CTMP and Ethylphenidate in Rat Nucleus Accumbens and Bed Nucleus of the Stria Terminalis

**DOI:** 10.3389/fpsyt.2018.00149

**Published:** 2018-05-28

**Authors:** Colin Davidson, Christopher A. R. Raby, Vincenzo Barrese, John Ramsey

**Affiliations:** ^1^Basic Medical Science, St George's University of London, London, United Kingdom; ^2^Pharmacy & Biomedical Sciences, University of Central Lancashire, Preston, United Kingdom; ^3^TICTAC Communications Ltd, St George's University of London, London, United Kingdom

**Keywords:** novel psychoactive substance, dopamine, noradrenaline, voltammetry, brain slice, adolescent

## Abstract

3,4-dichloromethylphenidate (3,4-CTMP) and ethylphenidate are new psychoactive substances and analogs of the attention deficit medication methylphenidate. Both drugs have been reported on online user fora to induce effects similar to cocaine. In the UK, 3,4-CTMP appeared on the drug market in 2013 and ethylphenidate has been sold since 2010. We aimed to explore the neurochemical effects of these drugs on brain dopamine and noradrenaline efflux. 3,4-CTMP and ethylphenidate, purchased from online vendors, were analyzed using gas chromatography and mass spectroscopy to confirm their identity. Drugs were then tested in adolescent male rat brain slices of the nucleus accumbens and stria terminalis for effects on dopamine and noradrenaline efflux respectively. Fast cyclic voltammetry was used to measure transmitter release. Methylphenidate (10 μM) increased evoked dopamine and noradrenaline efflux by 4- and 2-fold, respectively. 3,4-CTMP (0.1 and 1 μM) increased evoked dopamine and noradrenaline efflux by ~6-fold and 2-fold, respectively. Ethylphenidate (1 μM) doubled evoked dopamine and noradrenaline efflux in both cases. 3,4-CTMP's effect on dopamine efflux was greater than that of methylphenidate, but ethylphenidate appears to be a weaker dopamine transporter inhibitor. Experiments using the dopamine D_2_ antagonist haloperidol, the noradrenaline α_2_ receptor antagonist yohimbine, the dopamine transporter inhibitor GBR12909 and the noradrenaline transporter inhibitor desipramine confirmed that we were measuring dopamine in the accumbens and noradrenaline in the ventral BNST. All three psychostimulant drugs, through their effects on dopamine efflux, may have addictive liability although the effect of 3,4-CTMP on dopamine suggests that it might be most addictive and ethylphenidate least addictive.

## Introduction

Concern has arisen over the past few years about “legal highs” or new psychoactive substances (NPS). Some of these NPS produce similar effects to cocaine, amphetamine, or MDMA (Ecstasy) and have changed the landscape of the UK drug scene by offering drug users the opportunity to use drugs without the risk of prosecution. The most prominent NPS is mephedrone, which was banned in the UK in 2010. The number of legal highs being sold on the Internet is increasing year-on-year. Deluca et al. [1] carried out a European-wide web-mapping project to quickly identify NPS use in Europe. By monitoring drug fora and websites, which provide information on legal highs between the drug communities, the authors uncovered 414 substances/products. The number of NPS discovered is now thought to be closer to 1000 (EMCDDA).

**Figure 1:**
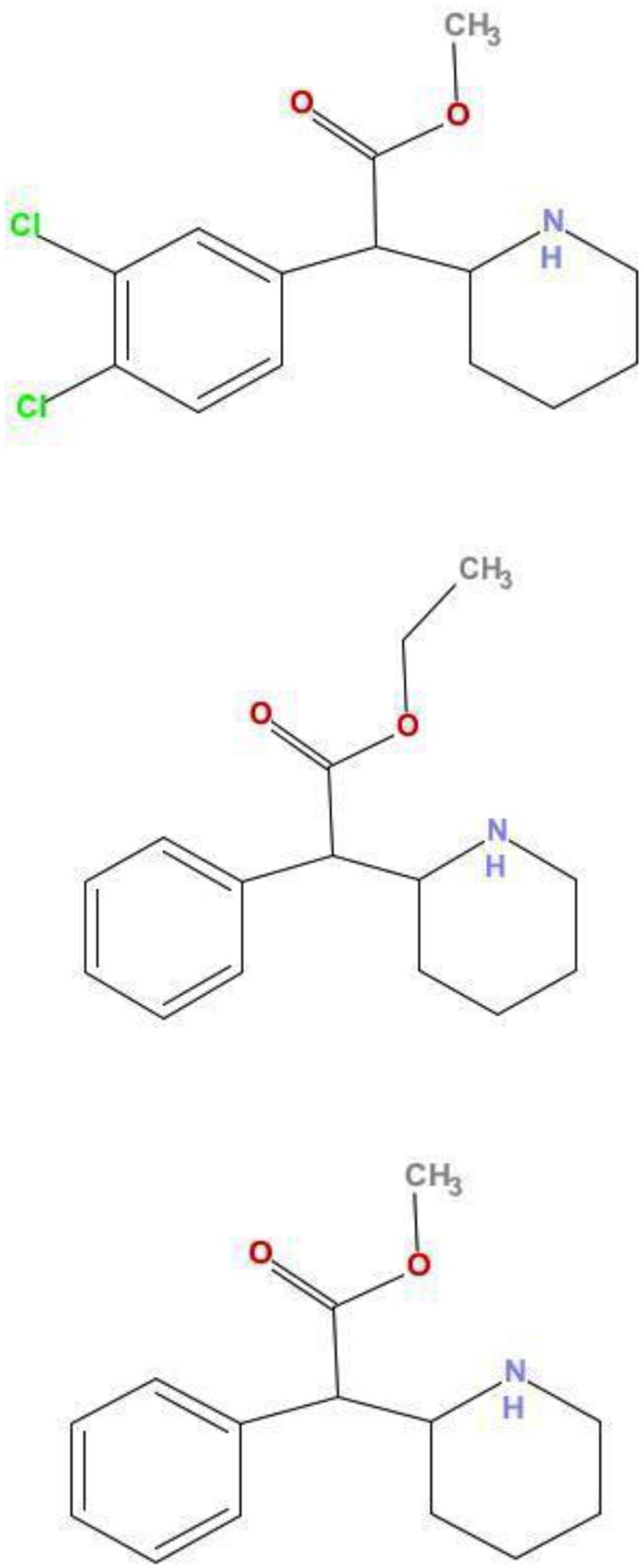
Comparison of structures of 3,4-CTMP, ethylphenidate, and methylphenidate. The chemical structures of 3,4-CTMP **(Top)** and ethylphenidate **(Middle)** are shown, along with that of methylphenidate **(Bottom)**. Their close structural relationship may be seen.

Many of these NPS have been associated with emergency room visits [2–4] and drug related deaths [5], it is therefore important to learn about the pharmacology of these substances, for which very little is known. We have recently identified 2 NPS from samples obtained from London dance club amnesty bins or samples purchased on the internet and examined these drugs, 3,4-CTMP and ethylphenidate, using neurochemical assays. 3,4-CTMP is an NPS that became available for purchase on the Internet in January 2013. Ethylphenidate is an NPS that has been sold since 2010. Both drugs are closely related to the attention deficit medication methylphenidate (Ritalin®; Figure 1).

Drugs such as cocaine, amphetamine, and MDMA increase dopamine concentration in the rodent nucleus accumbens [6, 7] making the accumbens an ideal location to investigate the addictive liability of NPS. Imaging studies of the human brain have also shown that increased dopamine levels in the striatum as a result of drug use are associated with the euphoric effects of drugs [8]. The nucleus accumbens and bed nucleus of the stria terminalis are both part of the extended amygdala [9], which is thought to integrate brain arousal-stress systems with hedonic processing systems. Stress induced reinstatement of some drugs of abuse in animal models appear to be partly dependent on the activation of noradrenaline in the BNST [10, 11]. The ventral BNST is highly innervated by noradrenergic neurons and has very few dopamine or 5-HT terminals [12–14]. This means measured transmitter release from the ventral BNST is directly attributable to noradrenaline. The ventral BNST is therefore ideal for studying drug effects on the noradrenergic system ([15–19]).

Here we examined 3,4-CTMP and ethylphenidate on dopamine and noradrenaline efflux in rat brain slices. We examined dopamine efflux as this would help us determined the likely abuse liability of these drugs. We examined noradrenaline efflux, as a drug that increases noradrenaline levels will likely be a vasoconstrictor and cause hypertension [20]. Thus, these data also give us some idea of the possible cardiotoxicity of the drug.

## Methods

The two NPS were found in samples from dance club amnesty bins in London (October—December 2012) and also from subsequent test purchases from internet sites. They were shown to be pure samples using gas chromatography with mass spectroscopy (GCMS) and nuclear magnetic resonance spectroscopy (NMR, data not shown). 3,4-CTMP was found in one amnesty bin sample while ethylphenidate was found in 27 samples often in combination with other active compounds. Three samples of each drug were purchased from different websites and all were found to be essentially pure.

### Animals and dissection

Brain slices used in all experiments were obtained from 8 week old male Wistar rats (Charles River Laboratories). Animals were housed 4 per cage, kept on a 12/12-h light/dark cycle and had food and water available *ad libitum*. Rats were killed by cervical dislocation, without anesthesia, as anesthetics are known to affect neurotransmitter levels, particularly monoamine neurotransmitters. Schedule 1 euthanasia procedures were done in accordance with the relevant regulations under the UK Animals (Scientific Procedures) Act 1986.

The brain was cut to ~+3 and −5 mm vs. bregma, which left a block including the nucleus accumbens and bed nucleus of the stria terminalis (BNST; [21]). Ice-cold aCSF was applied to the brain throughout the dissection. The posterior surface of the brain slice was glued to the chuck of a vibratome (Campden Instruments, Loughborough, UK). The brain and chuck were submerged in ice-cold aCSF and 400 μm coronal slices were taken to include the nucleus accumbens and BNST, ~+2.2 to −0.3 vs. bregma. Sections were then transferred to a slice saver and suspended on a plastic mesh in aCSF at room temperature, while continually bubbled with 95%O_2_/5%CO_2_.

### Chemicals and aCSF

Salts, glucose, haloperidol, yohimbine, GBR12909, and desipramine were bought from Sigma Aldrich (UK). Concentrations used were based on previous studies [15, 16]. Artificial cerebrospinal fluid (aCSF) was prepared daily. The composition of aCSF was: (mM): NaCl (126.0), KCl (2.0), KH_2_PO_4_ (1.4), MgSO_4_ (2.0), NaHCO_3_ (26.0), CaCl_2_ (2.4), (+)glucose (10.0), bubbled continually with 95% O_2_/5% CO_2_. 3,4-dichloromethylphenidate (3,4-CTMP) and ethylphenidate were initially dissolved in deionized water to 10 mM, then diluted with aCSF.

### Fast cyclic voltammetry

Fast cyclic voltammetry (FCV) is an electrochemical technique that takes advantage of the electroactive properties of the monoamine neurotransmitters. It can be used to quantitatively measure neurotransmitter concentrations, and how these concentrations differ in the presence of drugs, in real-time [22]. A triangular voltage waveform is applied to a carbon fiber microelectrode (Figure 4A), which oxidizes dopamine and noradrenaline at ~600 mV. Calibrations of electrodes in a known concentration of dopamine or noradrenaline allow the recorded Faradaic current to be converted into the relevant neurotransmitter concentration (Figure 4).

Using a Millar Voltammetric Analyser (PD Systems, West Molesey, UK) we sampled dopamine or noradrenaline levels at 8 Hz. Changes in the sampled signal were captured using a CED1401 micro3 analog-to-digital converter (Cambridge Electronic Design (CED), UK), displayed using Spike2 v7.1 data capturing software.

### Electrical stimulation protocol

Bipolar tungsten electrodes, with their tips 400 μm apart, were used to locally stimulate either the core of the nucleus accumbens or the ventral BNST (immediately below the anterior commissure). In most experiments pseudo-one pulse stimulation was used to avoid the activation of autoreceptors [23], which occurs ~500 ms after striatal dopamine release [24, 25]. A train of 10 × 1 ms 10 mA pulses at 100 Hz was applied every 5 min using a Neurolog NL800 stimulus isolator (Warner Instruments, Hamden, CT, USA) under computer control (Spike, CED). In experiments examining antagonists we used longer stimulation trains (10 pulses at 10 Hz, 900 ms train duration). Longer stimulation trains are useful in examining autoreceptor antagonists as described above. In the ventral BNST it has been found that stimulation frequencies of 10 or 20 Hz were best to see autoreceptor antagonist effects [16].

### Experimental protocol

To begin an experiment, slices were transferred from the slice saver to a laminar flow recording chamber that was supplied with aCSF via gravity feed, at a rate of 100 ml/h. Slices were left to equilibrate in the recording chamber for 30 min before starting stimulation of the slice, and the tips of both stimulating and recording electrodes were placed in the appropriate location in the brain to record monoamine release in eitherthe nucleus accumbens or ventral BNST. Recording took place from the beginning of this 30 min period as large spontaneous release events of dopamine can occur, which is indicative of poor slice health [26], and on such occasions (5–10%) the experiments were terminated.

### Carbon fiber microelectrodes

Carbon fiber microelectrodes were constructed by inserting a single carbon fiber (Goodfellow Cambridge Ltd, UK), 7 μm in diameter, into a 10 cm long borosilicate glass capillary tube. The capillary tube was then pulled using an electrode puller (P-30, Sutter instruments Co, USA) and the exposed carbon fiber was cut to ~70 μm under a microscope using a scalpel. Microelectrodes were backfilled with a saline solution before a length of copper wire was inserted into the end so it could be connected to the head-stage. A Ag/AgCl reference electrode and a steel wire auxiliary electrode were also connected to the head-stage and positioned within the recording chamber fluid, well away from the slice. Carbon fiber electrodes were calibrated using 5 or 10 μM dopamine or noradrenaline in aCSF. We also tested 3,4-CTMP and ethylphenidate in calibrations, to determine whether they would contribute to our signals (Figures 3C,D). Some drugs are electroactive themselves and/or can foul electrodes ([27]; Figure 4).

### Data acquisition and analysis

The current at the carbon electrode was sampled at 50,000 Hz by the computer and stored on one channel using Spike7. Another channel recorded changes at the dopamine or noradrenaline oxidation potential (600 mV). Peak height of transmitter efflux was calculated. An exponential curve was also fitted to the reuptake phase of the signal after each stimulation, using Spike7. This calculated the time-constants for the exponential decay phase of transmitter reuptake. The time-constant has been shown to be an appropriate method to accurately measure monoamine reuptake [28].

Dopamine or noradrenaline efflux was electrically evoked every 5 min and peak height was measured. Aggregate reuptake data are not shown as on some occasions, especially in the BNST and most often when drugs were present, the transmitter signal did not fall to basal levels in an exponential fashion, making modeling of reuptake difficult. In addition, it has been shown that peak height correlates well with reuptake effects [28] and so we only show peak height effects here. Baseline (before drug application) reuptake time-constants are given in the results section (Baseline Efflux Data). Once three steady baseline efflux events had been obtained, the drug was added for 60 min. See Figures 3A,B for example experiments. Data was converted to a percentage of mean baseline data for statistical analysis.

Statistical analysis of differences between the two drugs on evoked transmitter release was carried out using two-way ANOVA with Tukey's *post-hoc* multiple comparisons test (drug X concentration). The data using antagonists or GBR12909/desipramine were analyzed by one-way AONOVA. For all analysis we used the peak effect of the drugs at each concentration. Data is presented as mean ± standard error of the mean (SEM), with a significance level set at *p* < 0.05.

## Results

### Baseline efflux data

**Figure 2:**
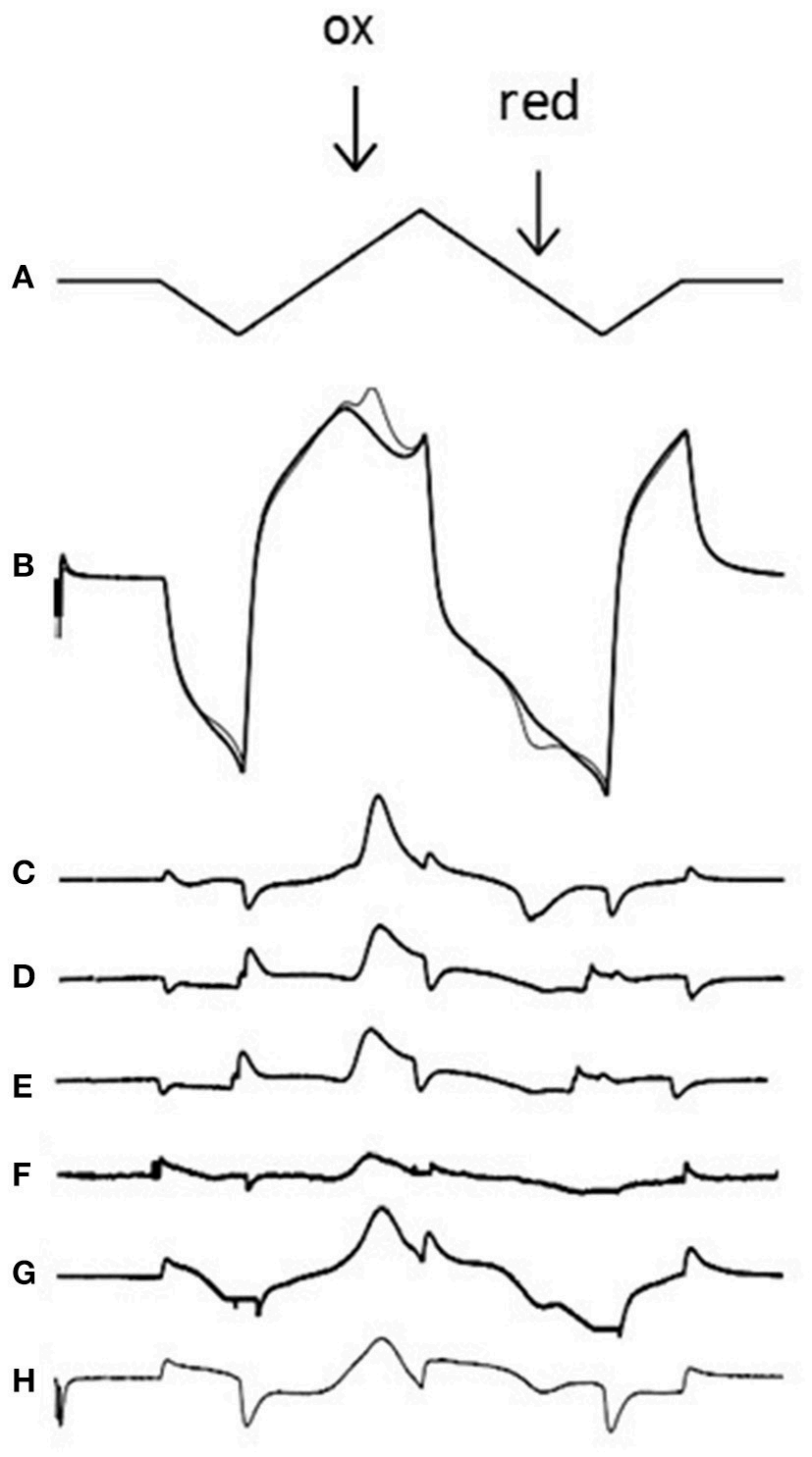
Voltammetric technique and voltammograms for exogenous and endogenous amines. **(A)** The applied voltage to the carbon electrode (0 to −1 to +1.4 to −1 and back to 0 V at 480 V/s which takes 20 ms). **(B)** The charging current at the electrode in the absence (thick line) and presence (thin line) of 5 μM dopamine; note the dopamine oxidation peak (ox; 0.6 V) where dopamine releases 2 electrons, creating a Faradaic current. Note also the reduction peak (red; −0.2 V) where dopamine-o-quinone is reduced back to dopamine, using 2 electrons. If one subtracts the signal in the presence of dopamine from the signal in the absence of dopamine one is left with a voltammogram **(C)**. Voltammograms are also shown for 5 μM noradrenaline **(D)**, stimulated dopamine in the accumbens **(E)**, and stimulated noradrenaline in the BNST **(F)**. The voltammogram from the apparent ethylphenidate (30 μM) evoked increase in basal accumbens dopamine (see Figure 3C) is shown in **(G)**, but the voltammogram from the calibration of the electrode in 30 μM ethylphenidate (see Figure 3D) reveals that this drug is electroactive **(H)**. In each case the oxidation peak is clearly seen. Note that we often get small non-biological artifacts when the voltage changes direction. Neither methylphenidate nor 3,4-CTMP were found to be electroactive (data not shown).

We used local electrical stimulation to evoke both dopamine and noradrenaline efflux in the accumbens and BNST respectively. Baseline (non-drug) dopamine efflux had a peak height of 419 ± 50 nM with decay phase time-constant of 1.23 ± 0.08 s (*n* = 66). Baseline noradrenaline efflux had a peak height of 169 ± 19 nM with decay phase time-constant of 1.84 ± 0.16 s (*n* = 43). See Figure 2 for example efflux events.

**Figure 3:**
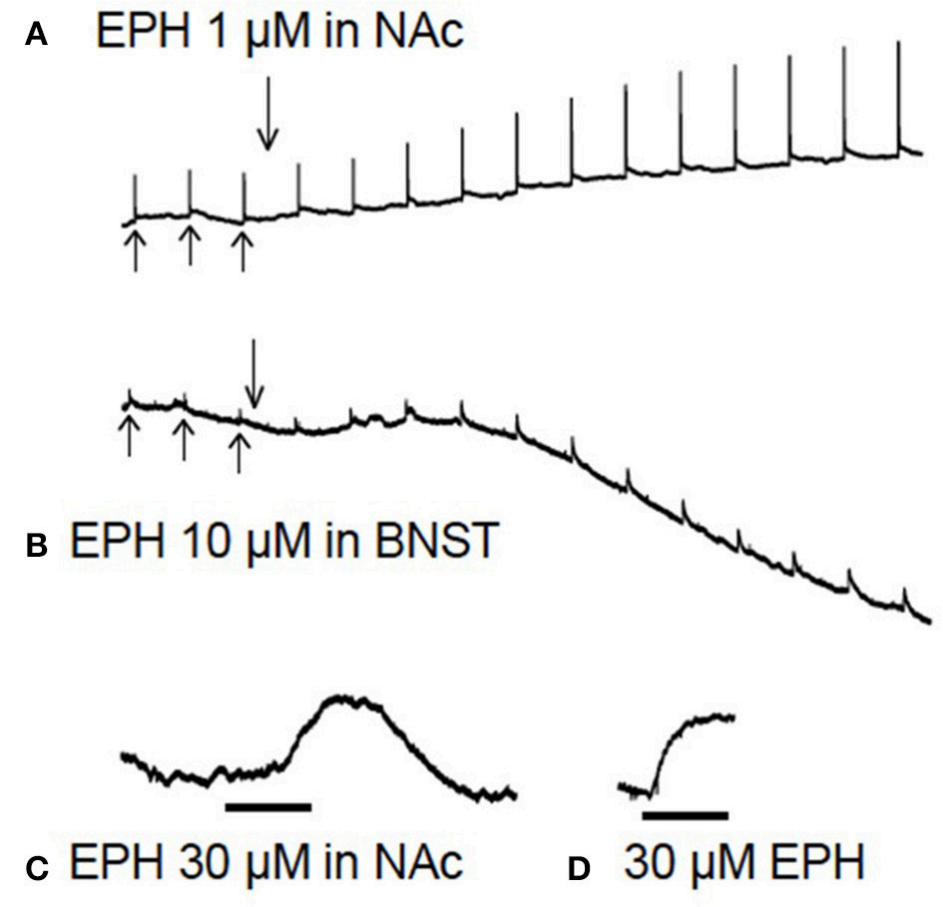
Raw data from brain slice experiments. In **(A,B)**, after 3 baseline electrical stimulations (upward arrows), the drug is added (downward arrow) for 60 min while stimulations continue every 5 min. In the lower traces **(C,D)** there are no electrical stimulations. **(A)** Shows evoked dopamine efflux in the nucleus accumbens (NAc) after application of 1 μM ethylphenidate (EPH), note the increase in electrically evoked dopamine. **(B)** Application of EPH (10 μM) in the bed nucleus of the stria terminalis (BNST), note the increase in stimulated noradrenaline efflux and the apparent increase in background levels of transmitter 10 min after drug application. **(C)** Application of 30 μM EPH (black bar = 10 min) in the NAc appeared to increase basal dopamine levels (there is no electrical stimulation in this trace), but on testing 30 μM EPH at the carbon electrode in a calibration it was found that this drug is electroactive **(D)**, although not nearly as electroactive as dopamine or noradrenaline.

**Figure 4:**
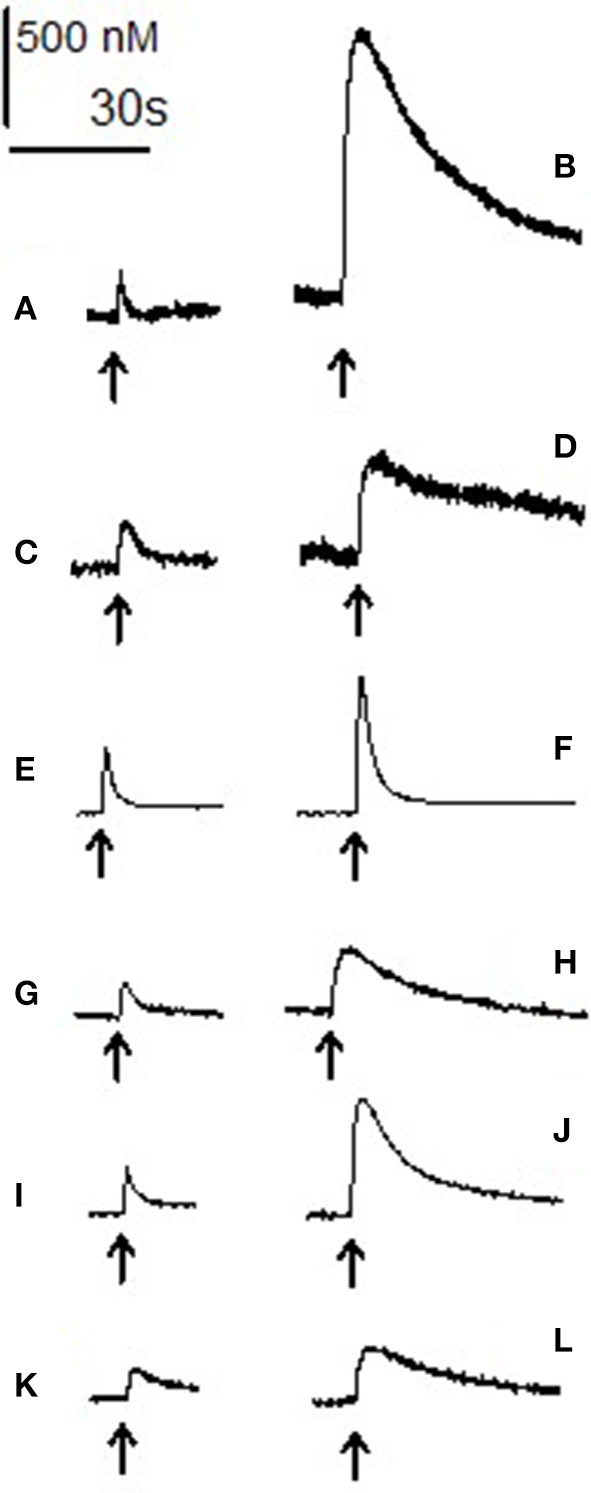
Electrically evoked dopamine and noradrenaline: raw data. Each trace shows the increase in amine level after electrical stimulation (arrow) and its subsequent reuptake in the absence (left) and presence (right) of the three psychostimulant drugs. **(A)** Baseline stimulation in accumbens and **(B)** stimulation after 60 min of 10 μM 3,4-CTMP. **(C)** Baseline evoked efflux in the BNST and **(D)** after 60 min of 10 μM 3,4-CTMP. **(E)** Baseline evoked efflux in the accumbens and **(F)** after 60 min of 10 μM ethylphenidate, **(G)** baseline evoked efflux in the BNST and **(H)** after 60 min of 10 μM ethylphenidate. **(I)** Baseline evoked efflux in the accumbens and **(J)** after 60 min of 10 μM methylphenidate, **(K)** baseline evoked efflux in the BNST and **(L)** after 60 min of 10 μM methylphenidate. All traces are scaled such that the bars denoting concentration and time are accurate for each trace. The 2 traces in each pair (**A** and **B; C** and **D; E** and **F; G** and **H; I** and **J; K** and **L**) were taken from the same brain slice.

### Effect of methylphenidate, 3,4-CTMP, and ethylphenidate on amine efflux in the accumbens

**Figure 5:**
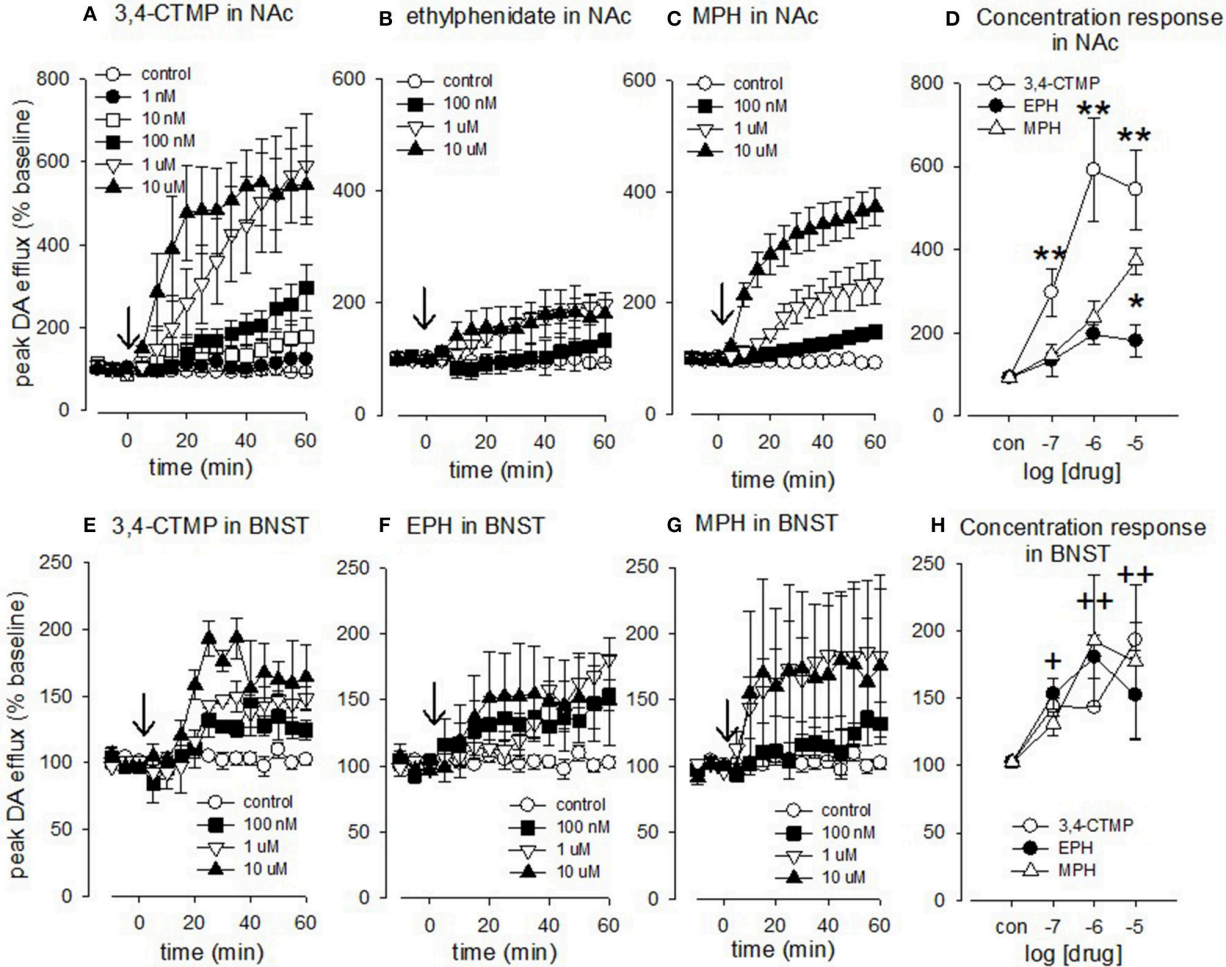
Effect of methylphenidate, 3,4-CTMP and ethylphenidate in the nucleus accumbens (NAc) and bed nucleus of the stria terminalis (BNST) slice. After 3 baseline electrical stimulations (10 pulses at 100 Hz) methylphenidate, 3,4-CTMP or ethylphenidate (EPH) was added at a single concentration (arrow). The drug was left on for 60 min and stimulations continued at 5 min intervals. **(A)** Effect of 3,4-CTMP in the accumbens. **(B)** Effect of ethylphenidate in the accumbens. **(C)** Effect of methylphenidate in the accumbens **(D**) Comparison of methylphenidate, 3,4-CTMP and ethylphenidate in the accumbens using a two-way ANOVA. Asterisks denote difference between drugs, **(E)** Effect of 3,4-CTMP in the BNST. **(F)** Effect of ethylphenidate in the BNST. **(G)** Effect of methylphenidate in the BNST. **(H)** Comparison of methylphenidate, 3,4-CTMP and ethylphenidate in the BNST using a two-way ANOVA; there was no significant difference between the three drugs, but there was a significant effect of drug concentrations as denoted by the cross (+). Values are means ± SEM, *n* = 3–8. ^*^p < 0.05, ^**^p < 0.005, ^+^*p* < 0.05, ^++^*p* < 0.005.

Using a two-way ANOVA we compared the effects of methylphenidate, 3.4-CTMP and ethylphenidate on electrically evoked dopamine efflux in the accumbens (Figures 5A–D). There was a main effect of drug [*F*_(2, 62)_ = 38.929, *p* < 0.001] a main effect of drug concentration [*F*_(3, 62)_ = 43.756, *P* < 0.001] and a drug X concentration interaction [*F*_(6, 62)_ = 8.67, *p* < 0.01]. Within the methylphenidate treated slices both 1 and 10 μM caused a significant increase in dopamine efflux (*p* < 0.05). Within the 3,4-CTMP treated slices, 100 nM caused an increase in evoked dopamine levels vs. controls (*p* < 0.05) while both 1 and 10 μM 3,4-CTMP increased evoked dopamine levels vs. both control and 100 nM (*p* < 0.05). Ethylphenidate did not significantly increase evoked dopamine levels at any concentration (all *p* > 0.1).

3,4-CTMP increased dopamine levels to a greater extent than both methylphenidate and ethylphenidate at 100 nM (*p* < 0.05 vs. both), 1 μM (*p* < 0.001 vs. both), and 10 μM (*p* < 0.001 vs. ethylphenidate and *p* < 0.05 vs. methylphenidate) while methylphenidate increased dopamine levels to a greater extent than ethylphenidate at 10 μM (*p* < 0.001).

### Effect of methylphenidate, 3,4-CTMP, and ethylphenidate on amine efflux in the BNST

Using a two-way ANOVA we compared the effects of methylphenidate, 3.4-CTMP and ethylphenidate on electrically evoked noradrenaline efflux in the BNST (Figures 5E–H). There was no main effect of drug [*F*_(2, 54)_ = 0.0781, *p* = 0.925] and no drug X concentration interaction [*F*_(6, 54)_ = 0.926, *p* = 0.486] but there was a main effect of drug concentration [*F*_(3, 54)_ = 13.933, *P* < 0.001, 10 μM (*p* < 0.001), 1 μM (*p* < 0.001), and 0.1 μM (*p* < 0.05)] all increased evoked noradrenaline efflux vs. controls.

### Characterization of amine efflux in accumbens and BNST

**Figure 6:**
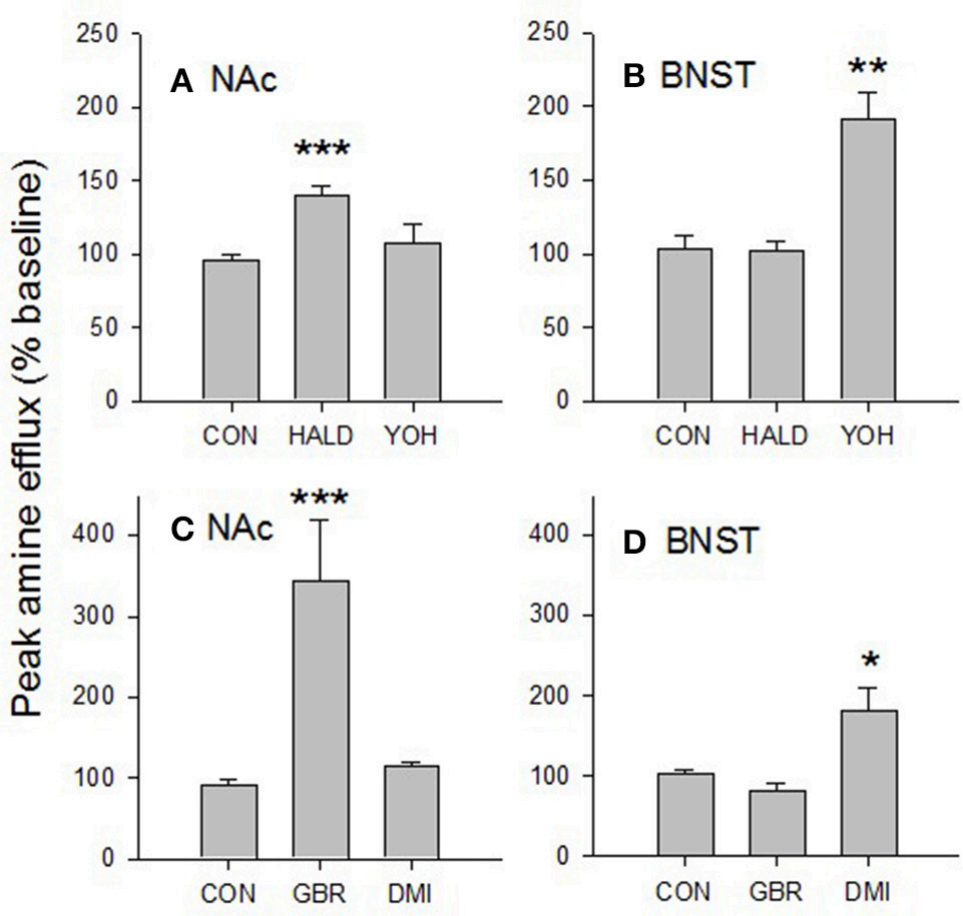
Characterization of amine released in the BNST and NAc. The dopamine D_2_ receptor antagonist, haloperidol (1 μM) increased electrically evoked amine efflux in the accumbens **(A)** but not the BNST. Similarly, the noradrenergic α_2_ receptor antagonist, yohimbine (1 μM), increased amine efflux in the BNST **(B)** but not the accumbens. The selective dopamine transporter inhibitor GBR12909 (200 nM) increased amine efflux in the accumbens **(C)** but not the BNST while the selective noradrenaline transporter inhibitor desipramine (50 nM) increased amine levels in the BNST **(D)** but not the accumbens. Taken together these data suggest that the amine we are measuring in the accumbens is dopamine, while that in the ventral BNST is noradrenaline. Values are means SEM taken at peak drug effect. Data were analyzed by one-way ANOVA. ^***^p < 0.001 vs. control; ^**^p < 0.01 vs. control; ^*^p < 0.05 vs. control. CON, control; HALD, haloperidol; YOH, yohimbine; GBR, GBR12909; DMI, desipramine. *N* = 2–8.

Using a one-way ANOVA we compared the effects of haloperidol and yohimbine in the accumbens and BNST. In the accumbens there was a significant effect of drug [*F*_(2, 18)_ = 10.598, *p* < 0.001; Figure 6A]. Tukey's test revealed that amine efflux was greater in the accumbens after haloperidol vs. control (*p* < 0.001) and was nearly greater after haloperidol vs. yohimbine (*p* = 0.056). There was no significant effect of yohimbine vs. control (*p* = 0.659).

In the BNST there was also a significant effect of drug [*F*_(2, 9)_ = 12.477, *p* < 0.005; Figure 6B]. Yohimbine increased amine efflux vs. haloperidol (*p* < 0.05) and control (*p* < 0.01). There was no significant difference in the effect of haloperidol vs. control (*p* = 0.999).

Again using a one-way ANOVA we tested the effects of GBR12909 and desipramine on amine efflux in the accumbens and BNST. In the accumbens there was a significant effect of drug [*F*_(2, 12)_ = 20.073, *p* < 0.001; Figure 6C]. Tukey's test revealed that GBR12909 had a significant effect on amine efflux vs. control (*p* < 0.001) and desipramine (*p* < 0.005). There was no significant effect of desipramine vs. control (*p* = 876).

In the BNST there was also a significant effect of drug [*F*_(2, 13)_ = 6.26, *p* < 0.005; Figure 6D]. Tukey's test showed that desipramine had a greater effect on amine efflux vs. control (*p* < 0.05) and GBR12909 (*p* < 0.05). There was no effect on amine efflux of GBR12909 vs. control (*p* = 0.813).

## Discussion

The majority of drugs of abuse increase dopamine efflux in the nucleus accumbens [6] by either directly acting on the dopaminergic terminals or indirectly at the ventral tegmental area. Both of the NPS studied here are structurally related to methylphenidate (Ritalin), a treatment for attention deficit hyperactivity disorder (ADHD). Methylphenidate increases dopamine levels by blocking the dopamine transporter (DAT) [29]. This potential for abuse has been exploited by legal high vendors, with methylphenidate's analogs 3,4-CTMP and ethylphenidate being offered for sale on many websites.

There is a paucity of published research surrounding 3,4-CTMP, which was developed and tested in the 1990's as a potential treatment for cocaine abuse [30]. It was suggested that some methylphenidate analogs could be used as a potential substitution therapy in the treatment of cocaine abuse, but 3,4-CTMP did not fall into this category. We have found 3,4-CTMP to dose-dependently increases electrically evoked dopamine release; 1 and 10 μM increased stimulated efflux ~6-fold. The most likely mechanism of action, through which 3,4-CTMP increases dopamine efflux, is by DAT inhibition. Methylphenidate has been shown to partially block the DAT [29], and a study using rotating disk electrode voltammetry in rat striatal suspensions proposed that halogenation of the aromatic ring of methylphenidate with chlorine increases a compounds affinity to the DAT [31]. In the case of 3,4-CTMP, this halogenation has occurred twice (at the 3′ and 4′ positions).

Previous work from this lab has examined the effects of cocaine on accumbens dopamine efflux [32, 33]. The present data suggest that 3,4-CTMP is slightly more potent than cocaine at increasing peak dopamine efflux in the rat accumbens slice. The potential increased potency of 3,4-CTMP vs. cocaine is supported by the findings of Deutsch et al. [30],who carried out ligand binding studies in rat striatal preparations to determine 3,4-CTMP's ability to inhibit [3H]WIN 35,428, a DAT inhibitor, as well as to look at the uptake of [3H]dopamine into rat striatal synaptosomes. They found that 3,4-CTMP was eight times more potent than methylphenidate at inhibiting WIN,35428 binding. Drug discrimination studies also suggest that 3,4-CTMP is much more potent than methylphenidate [34]. We have found 3,4-CTMP to increase dopamine levels more potently than methylphenidate (Figure 5D).

We also found 3,4-CTMP to increase electrically evoked noradrenaline efflux, with 10 μM resulting in an ~2-fold increase in the BNST. We have been unable to find any published data relating to the effect of 3,4-CTMP on noradrenaline levels, but methylphenidate does block the noradrenaline transporter (NET; [35]). Our data suggest that 3,4-CTMP may be have abuse liability, and that its effects are “cocaine-like” rather than “amphetamine-like,” as it increases stimulated dopamine release, but not basal (unstimulated) dopamine levels [36]. Similarly, we found no evidence for methylphenidate or ethylphendiate to cause reverse transport of dopamine (data not shown).

There is a lack of published research on the effects of ethylphenidate on monoamine release. Ethylphenidate was first identified in two overdose victims who had co-administered large quantities of methylphenidate and ethanol [37], which resulted in the formation of the previously unknown metabolite of these two substances, ethylphenidate. The formation of ethylphenidate, in rat liver preparations incubated with methylphenidate and ethanol, showed a carboxylase- dependant trans-esterification process [38] that is thought to be analogous to the formation of cocaethylene; the metabolite produced with co- administration of cocaine and ethanol [39]. The augmented effects of co-administration of methylphenidate and ethanol on mouse locomotion and brain levels of ethylphenidate have been examined [40, 41]. Co-administration of these drugs has also been tested in humans where ethylphenidate was shown to be produced along with increased subjective highs [42].

Internet drug fora have provided some useful information on the effects of ethylphenidate, with some reports suggesting that it produces a less jittery, more euphoric high than methylphenidate^1^^,^^2^. Additionally, multiple user reports confirm euphoria associated with ethylphenidate, as well as less locomotor stimulation, increased music appreciation and abstract thinking.

We found ethylphenidate to increases electrically evoked dopamine efflux by ~2-fold. The likely mechanism of action is by blocking the DAT; the same mechanism of action as methylphenidate [43]. In previous ligand binding studies, ethylphenidate has been shown to bind to the dopamine transporter with ~50% less binding potency than methylphenidate [29]. Ethylphenidate also increased locomotor activity in mice by 20% less than methylphenidate [44, 45]. Taken together these studies suggest that ethylphenidate is not as potent as methylphenidate at increasing dopamine levels, consistent with the present study (Figure 5D).

## Conclusion

This study explored the neurochemical profile of the NPS methylphenidate analogs 3,4-CTMP and ethylphenidate as well as the parent compound methylphenidate. 3,4-CTMP was more potent than methylphenidate, increasing dopamine release ~6-fold, presumably caused by blockade of the DAT. Ethylphenidate has a weaker effect, modestly increasing stimulated dopamine release by ~2-fold. All 3 drugs increased noradrenaline efflux ~2-fold. At the concentrations tested, no drug increased basal levels of dopamine or noradrenaline, that is, they did not cause reverse transport as one might expect with an amphetamine-like drug. The effects of each drug on dopamine indicate that they have the potential to be addictive, especially 3,4-CTMP.

## Ethics statement

This work was approved by the ethics committee at St George's University of London. Because only *in vitro* work was carried out and animals were killed by schedule 1 methods, no UK Home Office License was needed. Thus this work was done in accordance with the UK animal scientific procedures act.

## Author contributions

CD conceived of the work, undertook some experiments, did the statistics and wrote most of the manuscript. CR and VB undertook most of the experiments and helped analyse data. JR helped write themanuscript and conceived some of the work and analysed the NPS structures using mass spec.

### Conflict of interest statement

JR was employed by company TICTAC Communications Ltd. The other authors declare that the research was conducted in the absence of any commercial or financial relationships that could be construed as a potential conflict of interest.

## Footnotes

^1^Drugs Forum – legal high user blog. Available online at: http://www.drugs-forum.com/forum/showthread.php?t=137752 (Accessed July, 16 2013)

^2^Drugs Forum: Ethylphenidate. Available at: http://www.drugs-forum.com/forum/showthread.php?t=134578&page=2 (Accessed June, 05 2013)
